# RETRACTION: P4HA2 Promotes Epithelial‐to‐Mesenchymal Transition and Glioma Malignancy through the Collagen‐Dependent PI3K/AKT Pathway

**DOI:** 10.1155/jo/9783154

**Published:** 2026-03-22

**Authors:** Journal of Oncology

RETRACTION: J. Lin, L. Jiang, X. Wang, et al., “P4HA2 Promotes Epithelial‐to‐Mesenchymal Transition and Glioma Malignancy through the Collagen‐Dependent PI3K/AKT Pathway,” *Journal of Oncology*, 2021, 1406853, https://doi.org/10.1155/2021/1406853.

The above article, published online on 16 August 2021 in Wiley Online Library (https://wileyonlinelibrary.com), has been retracted by John Wiley & Sons Ltd. The retraction has been agreed due to multiple concerns identified with the figures, both directly and through PubPeer [1]. Specifically,

• The U251/p‐AKT bands in Figure 5e appear highly similar to the H460/mTOR/IP bands shown in Figure 1d.
• The U251/AKT bands in Figure 5e appear highly similar to the H157/Actin/Lysate bands shown in Figure 1d of [2].
• The U251/GAPDH bands in Figure 5e appear highly similar to the H460/Actin/Lysate bands shown in Figure 1d of [2].
• The U251/AKT bands in Figure 5e also appear highly similar to the Actin bands shown in Figure 4f of [3].
• The U87MG/AKT bands for sh‐P4HA2 and sh‐‐GFP + DHB appear highly similar to the first two U251/FBW7 bands shown in Figure 4f of [3].

The authors were unresponsive when asked to provide a response to the concerns. Following an assessment, the publisher has concluded that the results and conclusions can no longer be considered reliable, and the article is therefore retracted.

The authors did not respond to the retraction.
